# A study on using custom 3D-printed tongue bites for radiotherapy patients with oral tongue carcinoma

**DOI:** 10.1007/s13246-026-01725-3

**Published:** 2026-03-19

**Authors:** Muhammad Furqan Zulqurnain, Haris Arif, Misbah Batool, Nouman Amjad, Robin Hill

**Affiliations:** 1 Department of Physics and Applied Mathematics, Pakistan Institute of Engineering & Applied Sciences (PIEAS), Islamabad, Pakistan; 2 Medical Physics Department, Institute of Nuclear Medicine and Oncology (INMOL), Lahore, Pakistan; 3 Institute of Medical Physics, School of Physics, University of Sydney, NSW 2006 Australia; 4 Central Coast Cancer Centre, Gosford Hospital, Gosford, NSW 2250 Australia

**Keywords:** 3D tongue bite, 3D printing, Styrofoam tongue bite, Oral cancer, Tissue-equivalent, Biocompatible, Volumetric-modulated arc therapy (VMAT)

## Abstract

Oral cavity cancers are a debilitating form of head and neck cancer with high rates of mortality. Radiotherapy is one of the main forms of treatment but relies on minimizing doses that are delivered to organs at risk and considering any motion in the mouth. One solution is to use a block within the mouth which acts to reduce motion and decreases tissue heterogeneity. In this work, we developed a process for designing a customized tissue-equivalent 3D-printed tongue bite and evaluated its impact on the radiation treatment. Six patients with stage III or IV oral cancer were involved. Computed tomography (CT) images for each patient were acquired with currently used Styrofoam tongue bites within the mouth. The designs of 3D tongue bites were prepared using those CT images and then printed on an SLA printer using F80 resin which is a tissue-equivalent and biocompatible material. Secondary CT images were then acquired for each patient with the 3D tongue bites to have a dosimetric comparison. Volumetric-modulated arc therapy (VMAT) planning was carried out for individual patients on both CT images. Plan parameters, fractionation scheme and optimization priorities were all kept the same. The radiotherapy plans utilizing 3D tongue bites showed better PTV coverage and reduced D_max_ (*p* = 0.028). Doses to organs at risk (OARs) including brainstem, parotid glands and hard palate were also reduced (*p* < 0.028) except for the spinal cord (*p* > 0.05). The dose conformity and homogeneity were also improved (*p* = 0.028 and *p* = 0.044 respectively). All patients reported that the 3D tongue bites were soft, conformal to the oral cavity, comfortable and did not cause any gag reflex. We conclude that the 3D tongue bite is a useful utility that improves the treatment of patients with oral cancer.

## Introduction

Cancers of the oral cavity are one of the most common malignancies having high mortality rates. According to the International Agency for Research on Cancer (IARC) 2022 database, 389,846 cases of lip and oral cavity cancers were reported of which 188,438 were deaths. In Pakistan, a total of 10,181 fatalities were reported from a total of 15,915 cases [1]. It is the most prevalent site of origin for malignancy in the head and neck (H&N) region as it harbors carcinogens from alcohol, tobacco, and betel nuts. Malignant tumors of the oral cavity mostly occur at the anterior two-thirds of the tongue. Treatment may be carried out through surgery, chemotherapy, radiotherapy or a combination of the mentioned modalities. The cancer stage and the general health condition of the patient determines the optimal treatment strategy for the primary tumor [2, 3].

Due to the many clinical and geometric complexities of the oral cavity, external beam radiotherapy (EBRT) is delivered in the form of the intensity-modulated radiation therapy (IMRT) and volumetric-modulated arc treatment (VMAT) [4–6]. However, there are clinical complications in delivering the radiation treatment to the oral cavity. One to two weeks after receiving fractionated radiation therapy, the surrounding structures that are exposed to radiation usually experience radiation-induced side effects like xerostomia, taste loss and mucositis [7–9]. Moreover, the tongue, lips, and mandible are not static structures that have positional variations during computed tomography (CT) procedures and radiation treatments [10]. Another problem associated with the radiation therapy of tongue carcinoma is the underdosage of the superficial tongue tissue due to the tissue heterogeneities in the oral cavity and in-depth dose build-up. This results in an increased hotspot dose in the tumor volume and increased doses to the OARs [9].

Several measures have been taken to manage these issues with devices like oral corks, specialized stents and tongue displacement devices (TDDs). These fabrication devices reduce tissue heterogeneities leading to uniform dose distribution, reduced tongue mobility, and doses to OARs [8–13]. For instance, placing a special stent in mouth increases the distance between tongue and hard palate resulting in dose reduction to the hard palate. However medical conditions like trismus or a strong gag reflex can make it difficult for some patients as they cause discomfort. Most recently, three-dimensional silicone-printed bite blocks were tested that provided more immobilization, reduced doses to OARs and improved target coverage. These devices are not commonly utilized in clinical practice because of their complex manufacturing processes and high cost [9].

3D printing technology, initially introduced for production, has found its way into medicine and is increasingly more widely used in radiotherapy. This technology is facilitating the development of patient-specific treatment plans through the fabrication of patient-specific anatomical models, boluses, and various other implantable devices [14, 16]. 3D printing also facilitates individualized radiotherapy accessories, maximizing the treatment accuracy and dose distribution while sparing the normal tissue [14–16]. Yu-Ming Huang, et al. noticed that conventional techniques for immobilizing the teeth and tongue during radiation therapy for head and neck cancer were inadequate. They customized 3D-printed silicone bite blocks using SIL 28, a biocompatible material and investigated their clinical viability. These bite blocks that were manufactured using 3D technology were firm and elastic with the occlusal surface, immobilizing the tongue without triggering a gag response. The patients who used these bite blocks had lower doses to normal tissues and improved coverage of the planning target volume (PTV). The silicone 3D printed bite blocks proved useful, safe, and adaptable cutting edge radiotherapy technology for head and neck cancer [9]. However, silicone is a hard material to tolerate, and it is also expensive.

So, the current study aimed to customize patient specific 3D-printed tongue bite having properties of biocompatibility, tissue-equivalence, softness and cost-effectiveness. The study also evaluated the impact of the 3D tongue bite on radiation dose distribution in EBRT for oral tongue carcinoma. It involved two plans for individual patients: one with 3D tongue bite and another with Styrofoam tongue bite. The customization of 3D tongue bites was achieved by utilizing patient-specific anatomical data (DICOM images of oral cavities) and then through a 3D printer. Both groups underwent the planning process of Volumetric Modulated Arc Therapy (VMAT) treatment and various dosimetric parameters were evaluated for them.

## Methods

**Fig. 1:**
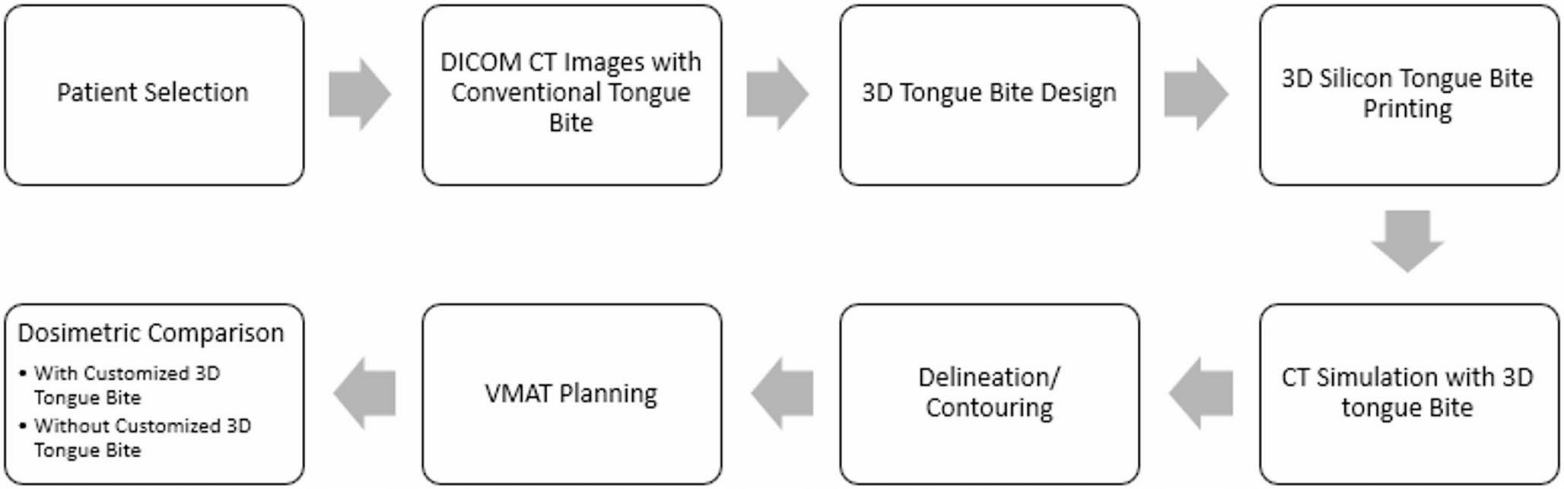
Clinical Flow of Radiotherapy Planning for Tongue Carcinoma with 3D-Printed Tongue Bites

The research was carried out at radiotherapy department, Institute of Nuclear Medicine and Oncology, Lahore (INMOL), Pakistan. The procedure followed in the current study is illustrated in Fig. 1.

### Patient selection

In the time span of the study, there were a total of 10 patients with tongue carcinoma in our radiotherapy department who were assessed for eligibility for this project. Four patients were not included due to their age or conditions with mouth bleeding. So, six tongue cancer patients were chosen for the study ranging in age from 40 to 60. The patients were in stages III and IV, with two patients having the tumor invading through the lymph nodes.

### Design of 3D tongue bite

At first, CT images of oral cavities with Styrofoam tongue bites were acquired. The Styrofoam pieces were of specific thickness to provide sufficient mouth opening (20–25 mm). Aquilion CT Simulator (Canon Medical Systems, Japan) was used with a slice thickness of 3 mm at 120 kVP and 190 mAs, Firstly, the individual patients were laid down on the couch in supine positions. To achieve proper immobilization, thermoplastic face masks and neck supports (Klarity, China) were utilized.

**Fig. 2:**
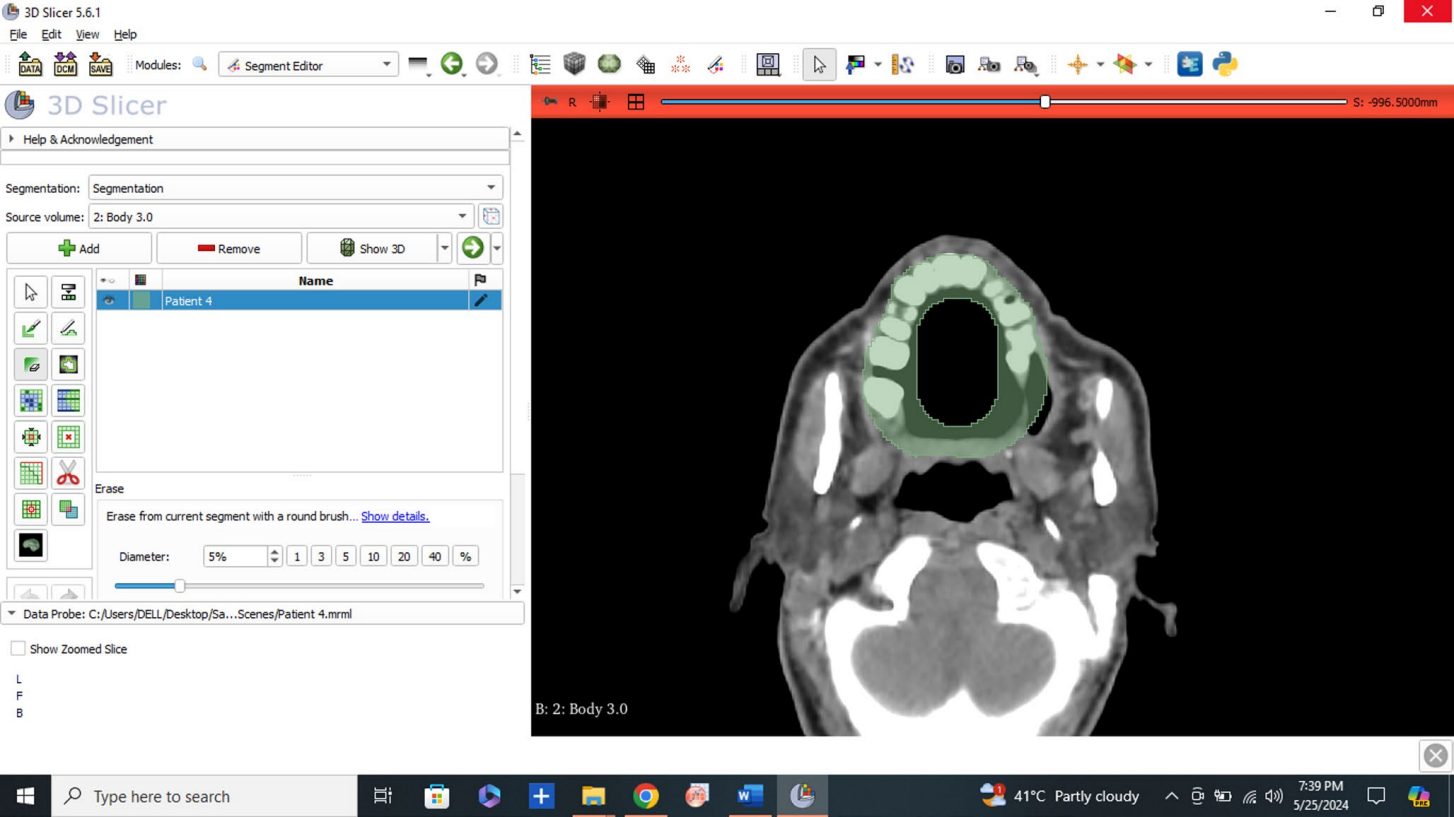
Slice by Slice Contouring of Upper and Lower Alveolus in 3D Slicer Software

Secondly, these images were imported into the open-source software 3D Slicer (version 5.6.1), where image segmentation was carried out using its segment editor module (Fig. 2).

**Fig. 3:**
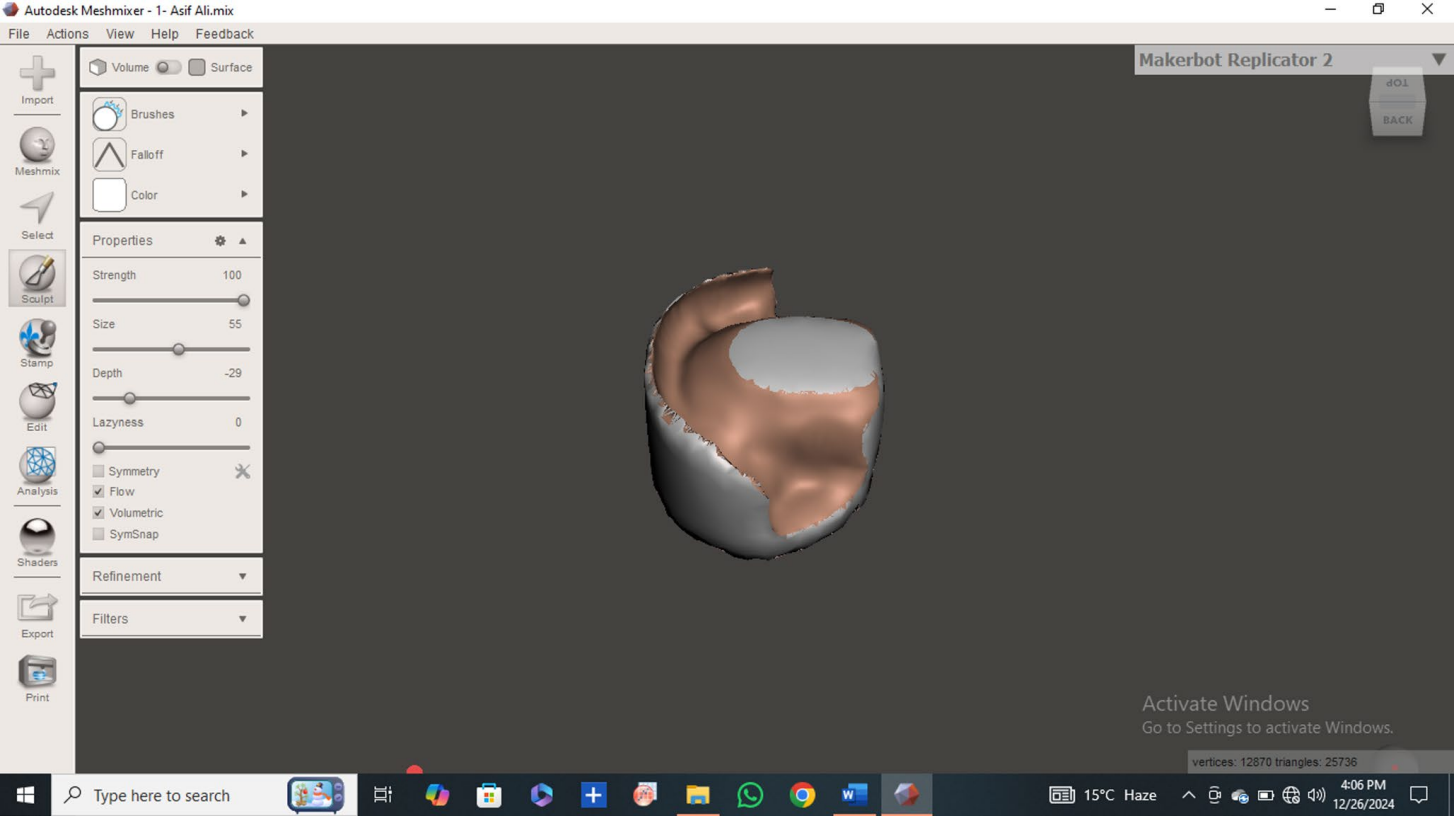
Final design of 3D tongue bite in Autodesk Meshmixer Software

Thirdly, this segmentation was exported to Autodesk Meshmixer software where the final design was prepared by Boolean subtraction (Fig. 3). Mostly, the designs in Meshmixer also require some smoothing which was also carried out for individual designs of 3D tongue bite.

**Fig. 4:**
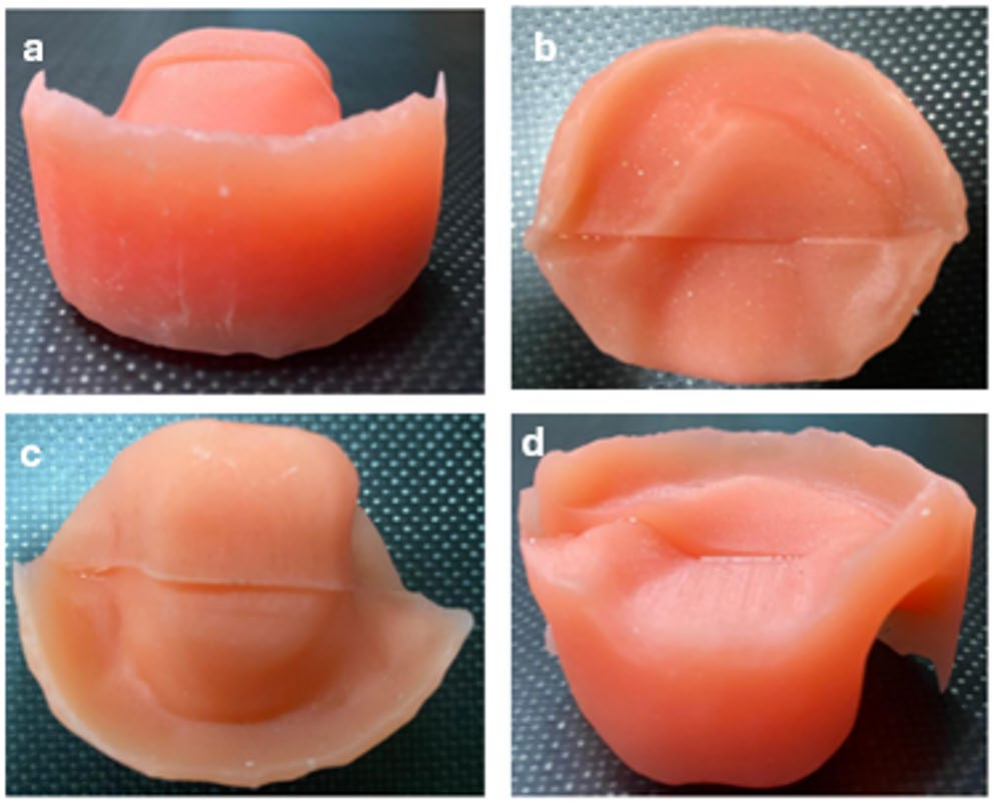
3D-Printed Tongue Bite

(*a*: Front view, *b*: bottom view, *c*: top view and *d*: back view)

### 3D tongue bite printing

The 3D printing was carried out on an Anycubic Photon Ultra (Anycubic, Shenzhen, China), which is a stereolithography (SLA) printer. Stereolithography (SLA) is a 3D printing technology that utilizes photopolymerization to solidify liquid resin into solid models. A stereolithography apparatus (SLA) machine points a laser beam on precise locations in a resin tray, curing the resin layer by layer from a digital 3D model. As each layer solidifies, it adheres to the layer beneath it, creating a very detailed and smooth 3D object [17].

A commercially available resin F80 (Resione, China) was used as the printing material. It is a tissue-equivalent and biocompatible material with high elasticity and excellent cold resistance, widely used for gingival modeling. The Hounsfield Units (HUs) and relative electron density of the resin were checked by CT scanning. The physical properties of the F80 resin are given in Table 1. The resin was preheated to 45 °C and then put in printer for printing process. The 3D printed tongue bite for one of the patients is shown in Fig. 4. The average printing time was 6 h per part [18]. As it was used as an intraoral device, each piece was washed in a methanol solution (90%) and then contained in a sterile polythene bag.

The printing process in carried out at 3D Buraq Studios (Model Town, Lahore, Pakistan). The resin cost Rs. 30/- per gram with an average weight of parts 150 g.

**Table 1:** Physical Properties of the printing material

Parameters	Measures
Mass Density Relative Electron Density HU Value	1.1 g/cm3 1.14^*^ 115^*^
Shore Hardness	50–60 A
Tensile Strength	3.8 MPa
Tear Strength	9.75 KN/m
Elongation at Break	159%
Viscosity (25 °C)	2300 mPa.s

^*^ Checked clinically

### Treatment planning

Patients were rescanned employing 3D tongue bites in mouth and two treatment plans were prepared for each patient, one with 3D printed tongue bite and one with the Styrofoam-based bite blocks. All the targets and OARs were delineated by the same radiation oncologist. The clinical target volume (CTV-HR) was defined at 0.5 cm margins from the gross tumor volume (GTV-High Risk) and the planning target volume (PTV-HR) was defined by 0.7 cm margins from CTV-HR. The nodal volumes were also delineated. The specific OARs included the brainstem, spinal cord, parotid glands and hard palate. Dose objectives for the targets and dose constraints for OARs were also advised by the oncologist and are given in Table 2.

PTV-HR was prescribed to be given 69.96 Gy in 33 fractions whereas PTV-IR was prescribed 59.4 Gy in same number of fractions. VMAT plans were generated using the Eclipse treatment planning system (Version 15.6.04, Varian Medical Systems, USA) with two and half arcs geometry. For optimization, this system makes use of the Photon Optimizer (PO) algorithm (version 15.6.04). Following optimization, the AcurosXB (AXB, version 15.6.04) algorithm was used for dose calculation with a grid size of 2.5 mm. To keep the planning consistent between two plans, the dose-volume constraints and their priorities were kept same during individual optimization.

Patients were delivered the radiotherapy holding 3D printed tongue bites in their mouth. Patient specific quality assurance (PSQA) for the patient plans was performed using portal dosimetry with a gamma analysis criterion of 3%/3 mm. Moreover, prior to every treatment the fit of the tongue bite in the patient was checked using kilovoltage cone-beam computed tomography (kV CBCT).

**Table 2:** Dose objectives for target and OARs

Organs	Dose limit
PTV-HR PTV-IR Brainstem Spinal Cord Parotid Glands Hard Palate	69.96 Gy 59.4 Gy D_max_ < 54 Gy D_max_ < 45 Gy Bilateral Mean < 25 Gy D_mean_ < 40 Gy

**Table 3:** Dosimetric comparison between plans with and without tongue bites

Parameters	With Tongue Bite (Mean Values)	Without Tongue bite (Mean Values)	|%Difference|	*p*-value
PTV (D_max,_ Gy)	76.16	79.34	4.0	0.028^a^
Spinal Cord (D_max_, Gy)	37.75	39.18	3.7	0.116^b^
Brain Stem (D_max_, Gy)	36.79	38.99	5.6	0.028^a^
Parotid-R (D_mean_, Gy)	22.65	25.26	10.0	0.028^a^
Parotid-L (D_mean_, Gy)	23.56	25.82	8.8	0.028^a^
Hard Palate (D_mean_, Gy)	7.64	11.93	35.9	0.028^a^
HI	0.06	0.09	33.3	0.044^a^
CI	1.27	1.35	5.9	0.028^a^
MUs	732	792.83	7.7	0.028^a^

^a^ Statistically Significant; ^b^ Statistically Insignificant

### Plan evaluation

The dosimetric evaluation for both plans were carried out over several parameters, including maximum dose to target, dose conformity and homogeneity, dose to critical structures and dose-volume histograms (DVHs) to compare target volume coverage and doses to OARs. In addition, several other parameters were also reviewed for both sets of plans. These were the tumor coverage factors (TCF), the monitor units (MUs) and the modulation factors (MFs) were also recorded for both plans. The conformity index was defined as [19]:

1 $$CI={V}_{PTV}\times\frac{{V}_{TV}}{{{TV}_{PV}}^{2}}$$

where ${V}_{PTV}$ is volume of PTV-HR, ${V}_{TV}$is the treated volume and ${TV}_{PV}$ is volume of ${V}_{PTV}$ within the ${V}_{TV}$. The homogeneity index was defined as [20]:

2 $$HI=\frac{{D}_{2\%}-{D}_{98\%}}{{D}_{50\%}}$$

where ${D}_{2\%}$ is the dose to 2% of PTV-HR volume, ${D}_{98\%}$ is the dose to 98% of PTV-HR volume and ${D}_{50\%}$ is dose to 50% of PTV-HR volume.

The tumor coverage factor was defined as:

3 $$TCF=\frac{VolumeofPTVrecievingreferencedose}{TotalVolumeofPTV}$$

where 95% of the prescribed dose is used as the reference dose.

The Modulation factor is a measure of the total modulation of the beams and is defined as:

4 $$MF=\frac{MonitorUnits}{DoseperFraction\left(cGy\right)}$$

### Statistical analysis

For small data sets that do not follow the normal distribution, non-parametric tests are applied. As our groups were dependent, the Wilcoxon signed-rank test was carried out using SPSS (Version 20, IBM Corporation, New York, United States) and the p-values were calculated. P-value < 0.05 was significant.

### Ethical statement

As the study involves human subjects, so the ethical approval was obtained from the Ethical Review Committee, INMOL with an approval number INMOL-27. Moreover, the consent from each patient was acquired after explaining to the patient the study methodology and purpose.

## Results

**Fig. 5:**
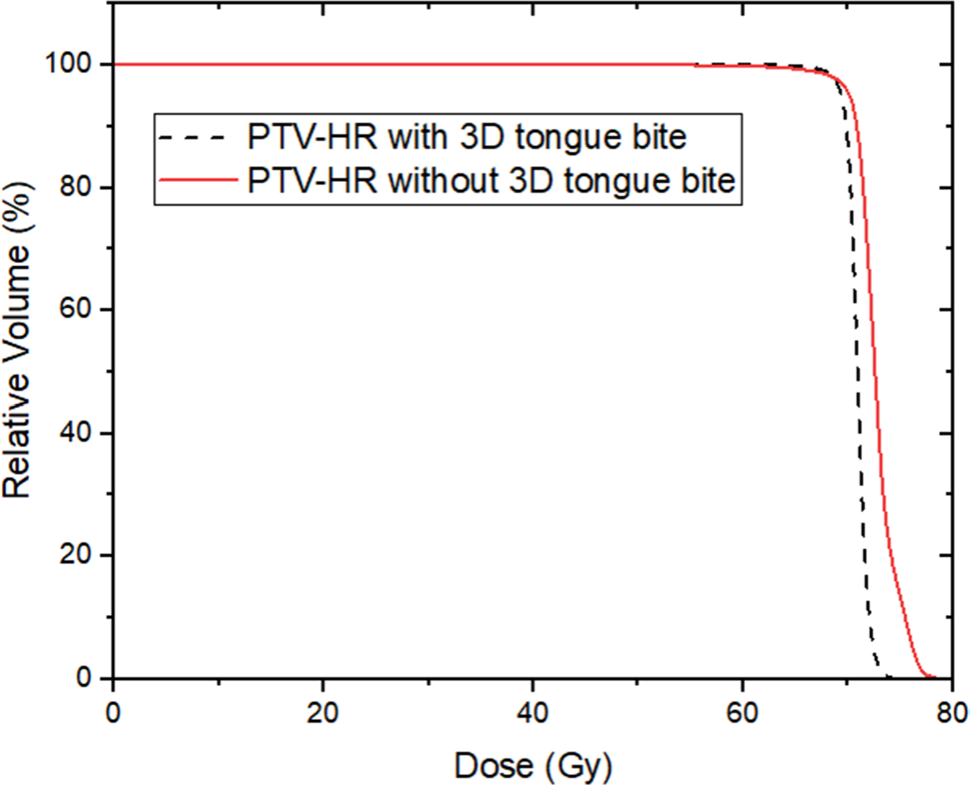
Average DVH of PTV-HR with and without 3D tongue bite

**Fig. 6:**
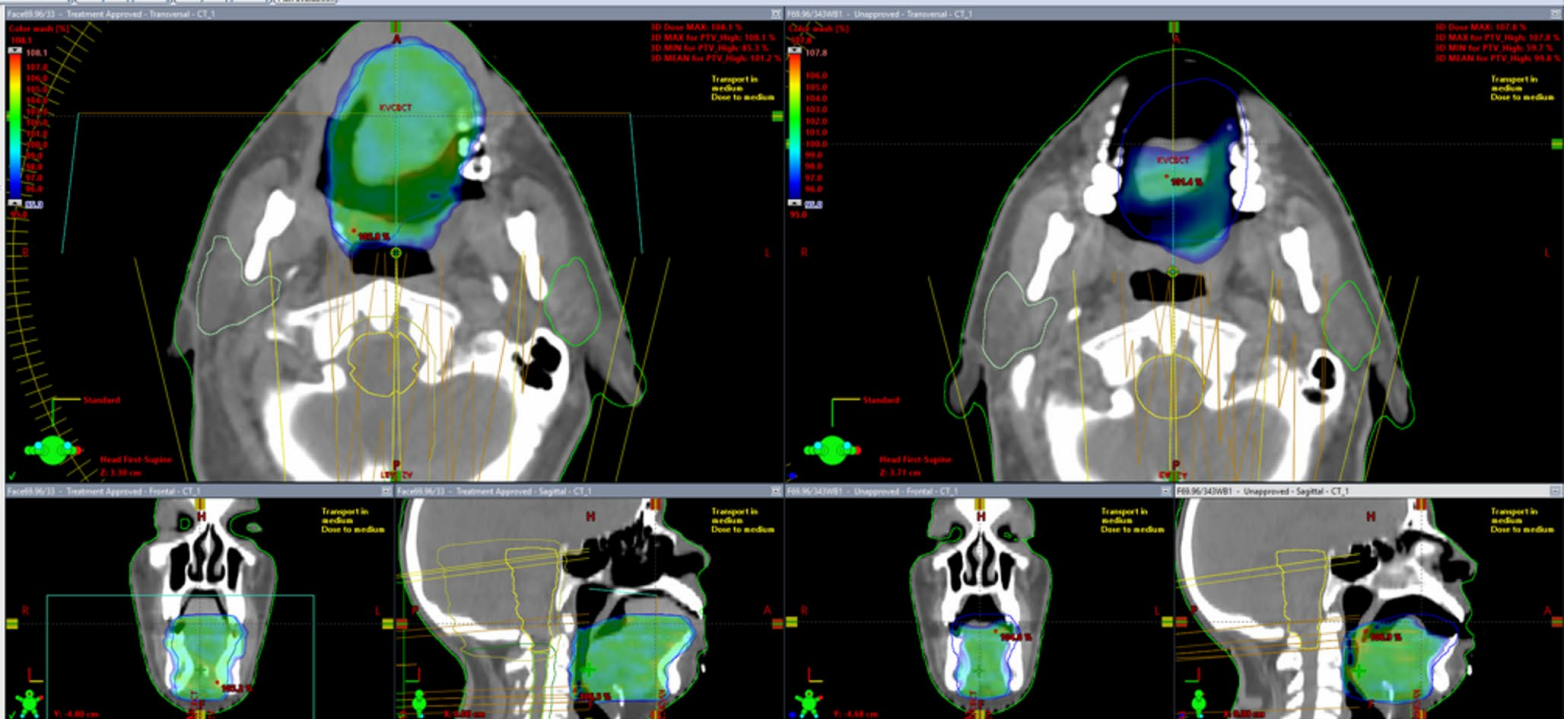
Target coverage comparison with missing target shown on the right side in the plan without tongue bite

**Fig. 7:**
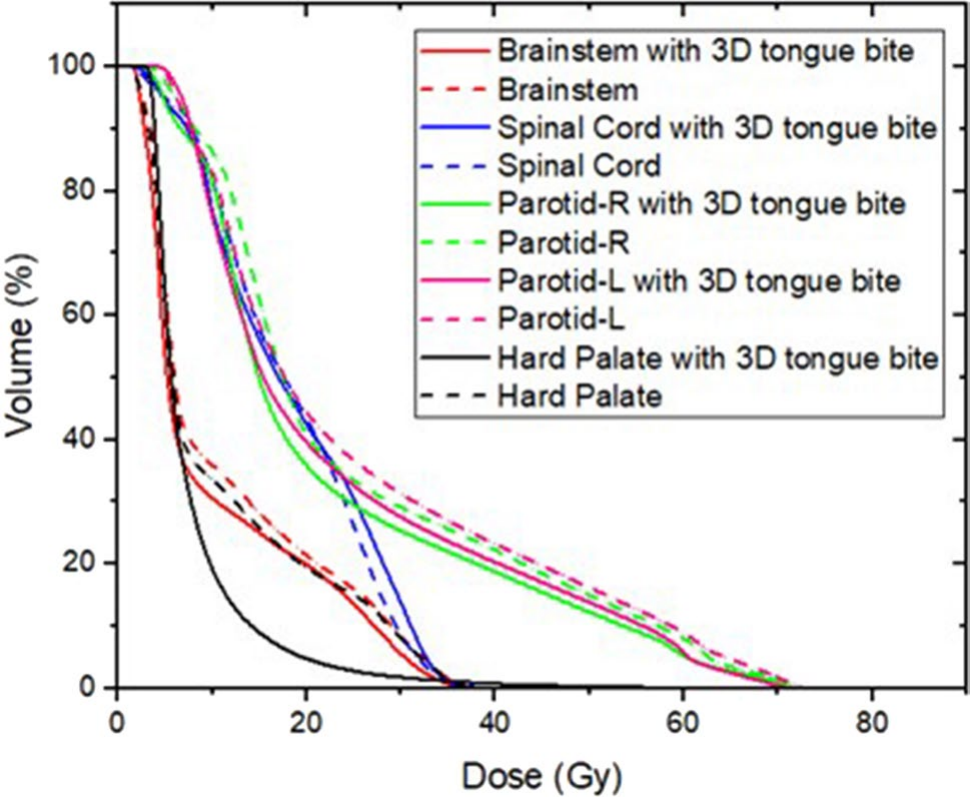
The average DVHs for the OARs with and without the 3D printed tongue bite

The VMAT plans for each patient with and without 3D tongue bite were dosimetrically evaluated based on D_max_, TCF, CI, HI, MUs and the doses to the OARs. The QA passing criteria (%) was 95.1 ± 2.3 (92.3–96.8) for plans with 3D tongue bite and 95.8 ± 1.9 (92.6–96.9) for plans with conventional tongue bite. A summary of the mean data is given in Table 3. The average DVHs for PTV-HR and OARs are shown in Figs. 5, 6 and 7 respectively. The TCF values in both plans were very similar (TCF = 0.99, *p* > 0.05), also can be seen in the average DVH of PTV-HR (Fig. 5). The reason is the same plan normalization, 98% isodose curve covering 98% of target volume. The average D_max_ to PTV-HR was 76.16 Gy for the plan utilizing tongue bite and 79.34 Gy for the plan with conventional tongue bite, respectively (*p* = 0.028).

The average values of CIs were found to be 1.27 and 1.35 for the plans with and without tongue bites respectively. On the other hand, the HIs were 0.06 and 0.09, respectively. The reduction in both indices was statistically significant (*p* = 0.028 for CIs and *p* = 0.044 for HIs). The average monitor units recorded were 732 for plans with 3D tongue bites and 792.83 for conventional bites respectively (*p* = 0.028). Figure 5presents scenario in which both plans are normalized at 100% volume covered by 95% isodose curve that clearly shows the missing target.

Plans utilizing 3D customized tongue bites showed reduced OAR doses. For brainstem, the dose reduced from 38.99 Gy to 36.79 Gy on average (*p* = 0.028). For spinal cord, average DVH of plans utilizing 3D tongue bites showed a merge or an overlapping behavior with others and no significant differences were seen (*p* = 0.116). The reason is the distance of spinal cord from the oral cavity. So, the effects of tongue bite can only be seen inside or near to the oral cavity. The D_mean_ to parotid-R reduced from 25.26 Gy to 22.65 Gy (*p* = 0.028), on average and for parotid-L, the dose reduced from 25.82 Gy to 23.56 Gy (*p* = 0.028), on average. Two patients had the parotid glands included in the target volume, so the dose limits couldn’t be adhered to them and mean value for parotid dose was calculated excluding these two patients in the results. The hard palate showed the greatest decrease in dose for the plans employing 3D tongue bites. On average, the dose reduced from 11.93 Gy to 7.64 Gy (*p* = 0.028) with mean reduction of 35.91%.

The 3D tongue bites were conformal inside the oral cavity, ensuring the patient comfort and it provided sufficient mouth opening of 20–25 mm. All the patients found the tongue bite soft and flexible without any gag reflex or other complexity observed.

## Discussion 

Oral Cavity tumors are the sites with much tissue inhomogeneities, tissue-air, bone-air, and tissue-bone. These interfaces cause rapid dose gradients and formation of hotspots. Moreover, the dose build-up property of high energy photon beams either underdose the superficial target or lead to the delivery of greater number of MUs resulting again in hotspots and normal tissue radiation-induced toxicities. In the radiotherapy of tongue carcinomas, efforts have been made to reduce the dose to OARs, attain the maximum target coverage and to lessen the inter- and intra-fractional motion during the radiotherapy. Several positioning stents and similar devices are being crafted and used for oral fixation and normal tissue sparing. Generally, all H&N cases employ the use of these fabricate devices.

B. Bø et al. [12] utilized the oral stents for increasing the distance between tongue and hard palate that resulted in dose reduction to hard palate so that mucositis can be avoided. Seongmoon Jung et al. [13] used several positioning devices to minimize the inter fractional motion and positional variations in tongue. For the patients not able to hold the stents, Whoon Jong Kil et al. proposed an alternate method of sticking-out tongue for dose reduction [21]. Chae-Seon Hong et al. [11] used 3D printer for the fabrication of tongue-displacement devices to reduce the dose to oral cavity. 3D-printed silicon bite blocks were designed, and their impact was studied by Yu-Ming Huang et al. It enhanced the target coverage and better normal tissue sparing [9].

In this present study, 3D tongue bites were crafted and their effects on radiation therapy were studied. In the first part of the study, 3D tongue bites were customized using CT images of oral cavities with a tissue equivalent and a biocompatible gum resin. The aim was to design soft and patient specific tongue bites that are patient comfortable. Moreover, it should provide sufficient mouth opening, depress and fix the tongue efficiently and fill the oral cavity well to attain maximum tissue homogeneity. In the second part of the study, a dosimetric comparison was made between the plans utilizing a 3D tongue bite and the plans with Styrofoam blocks.

The 3D tongue bites fit well inside the oral cavity, provided sufficient mouth opening, offering patient comfort with gag reflex reported. The tongue fixation using similar fabricated devices was confirmed by several studies [13, 22]. The dosimetric comparison also showed improved results. Plans with 3D bites showed a reduction in D_max_ (*p* = 0.028) and improved PTV coverage. The dose to PTV-HR reduced from 79.34 Gy to 76.16 Gy, on average. Except for the spinal cord (*p* > 0.05), doses for organs at risk (OARs) such as the brainstem, parotid glands, and hard palate were also reduced (*p* < 0.028). Hard Palate had the highest reduction with a mean reduction of 33%. Both homogeneity and the conformity showed improvements (*p* = 0.028 and *p* = 0.044, respectively). Additionally, there was also a decrease in the mean number of monitor units from 792.83 to 732, (*p* = 0.028).

In the available time span, we could only find six patients with overall good health conditions. The patients with older age and bleeding in cavity could not hold the tongue bites.

## Conclusion

 This was the first study to customize 3D printed bite block using resin. The current study concludes that the plans utilizing the 3D-printed tongue bites are superior based on decreased D_max_, OARs doses, modulation factor and greater target coverage. The future research may require more data points however, the current results are believed to be valid.
