# Correction to: A study on using custom 3D-printed tongue bites for radiotherapy patients with oral tongue carcinoma

**DOI:** 10.1007/s13246-026-01748-w

**Published:** 2026-05-26

**Authors:** Muhammad Furqan Zulqurnain, Haris Arif, Misbah Batool, Nouman Amjad, Robin Hill

**Affiliations:** 1 Department of Physics and Applied Mathematics, Pakistan Institute of Engineering & Applied Sciences (PIEAS), Islamabad, Pakistan; 2 Medical Physics Department, Institute of Nuclear Medicine and Oncology (INMOL), Lahore, Pakistan; 3 Institute of Medical Physics, School of Physics, University of Sydney, Sydney, NSW 2006 Australia; 4 Central Coast Cancer Centre, Gosford Hospital, Gosford, NSW 2250 Australia

**Correction to: Physical and Engineering Sciences in Medicine** 10.1007/s13246-026-01725-3

In this article the author’s name Muhammad Furqan Zulqurnain was incorrectly written as Muhmmad Furqan Zulqurnain.

The original article has been corrected.

